# A New *O*-Terpenoidal Coumarin from *Clausena anisum-olens* Merr.

**DOI:** 10.3390/molecules14020771

**Published:** 2009-02-13

**Authors:** Yun-Song Wang, Hong-Yi Xu, Da-Xiang Wang, Jing-Hua Yang

**Affiliations:** 1 Key Laboratory of Medicinal Chemistry for Natural Resource, Ministry of Education, School of Chemical Science and Technology, Yunnan University, Kunming 650091, P.R. China; E-mails: wangys@ynu.edu.cn (Y.-S.W.); yangjh@ynu.edu.cn (J-H.Y.); 2 Department of Public Security of Yunnan Province, Kunming 650031, P. R. China; E-mails: hongyix@sina.com (H-Y. X.)

**Keywords:** Rutaceae, *Clausena anisum-olens* Merr., Hekumarone, Monoterpenoid coumarins.

## Abstract

A new *O*-terpenoidal coumarin **1**, named hekumarone, was isolated from the leaves and twigs of *Clausena anisum-olens* Merr. (Rutaceae) collected in Hekou County in Yunnan Province, P. R. China. Structure elucidation and unambiguous NMR assignments for the title compound was carried out on the basis of 1D and 2D NMR experiments.

## Introduction

The plants of the Rutaceae family are well-known to contain structurally diverse and biologically active coumarins [1,2,3,4,5,6]. Within this family, the plants of the genus *Clausena* are shrubs, widely distributed in South and Southeast Asia and many species are used in Chinese folk medicine for various indications [7]. Previous phytochemical studies have indicated that *Clausena* species are the rich sources of carbazole alkaloids and coumarins [4,5,6,8,9,10,6,8]. *Clausena anisum-olens* Merr. is a shrub that both grows wild and is cultivated from Philippines and South China through Southeast Asia. The aerial parts of this plant have been used for the treatment of dysentery and arthritis [7]. Our previous phytochemical studies on *C. anisum-olens* Merr., collected in Hekou County in Yunnan Province, P. R. China, resulted in the isolation of new cyclic peptides and monoterpenoid coumarins [11,12,13]. In our ongoing search for bioactive compounds from this medicinally important genus, a new *O*-terpenoidal coumarin **1**, which we have named hekumarone, was isolated from *C. anisum-olens* Merr. In this report, we describe the isolation and structure elucidation of **1** using spectroscopic data analysis, including 1D and 2D NMR techniques (COSY, HSQC and HMBC). 

## Results and Discussion

The powdered plant material of *Clausena anisum-olens* Merr. (22.5 kg) was repeatedly extracted with EtOH at room temperature. The extract was then concentrated under reduced pressure to give a brown syrup, which was suspended in water and successively partitioned with petroleum ether, ethyl acetate (EtOAc) and *n*-butanol (*n*-BuOH). The EtOAc fraction was subjected to silica gel column chromatography, Pharmadex LH-20 and RP C-18 to yield compound **1**, which was isolated as a light yellow oil, ${\left[\alpha \right]}_{\text{D}}^{18.8}$ – 6.67 (*c* 0.75, CH_3_OH). The molecular formula C_20_H_18_O_7_ deduced from its ^13^C-NMR DEPT spectrum and HR-ESI-MS, which exhibited a molecular ion at *m/z* 369.1000 [M-1] ^+^, indicated twelve degrees of unsaturation. Strong UV bands at λ_max_ 256 and 319 and an IR band at 1,730 cm^-1^ indicated the presence of a 7, 8-dioxygenated coumarin [5]. 

**Figure 1:**
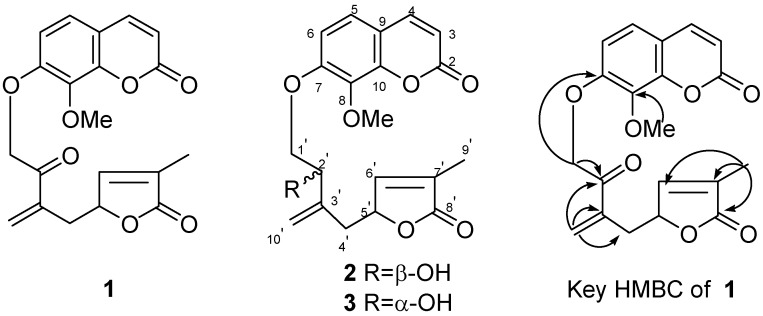
Structures of compounds **1**- **3** and key HMBC correlations of **1**.

The ^1^H-NMR spectrum (Table 1) showed resonances for two pairs of typical AB system characteristic doublets at δ_H_ 6.32 and 7.64 (each *J* = 9.5 Hz) and δ_H_ 6.98 and 7.20 (each *J* = 8.6 Hz), corresponding to H-3, H-4, H-5 and H-6 in the coumarin nucleus. Additional signals in the spectrum were consistent with three olefinic protons δ 6.37, 6.09 (each1H, s) and 7.01(1H, d, *J* =1.6 Hz), one oxymethylene group δ 5.57 (2H, s), two methylene protons δ 2.80, 2.62 and one olefinic methyl δ 1.77 could be seen.

**Table 1:** The ^1^H- and ^13^C-NMR data for compounds **1**-**3** (δ in ppm, *J* in Hz)*.

No.	1		2		3	
	*δ*_H_	*δ*_C_	*δ*_H_	*δ*_C_	*δ*_H_	*δ*_C_
2	/	159.2 (s)	/	160.4 (s)	/	160.4 (s)
3	6.32 (d,9.5)	112.7 (d)	6.33 (d, 9.8)	113.6 (d)	6.33 (d, 9.8 )	113.6 (d)
4	7.64 (d,9.5)	142.9 (d)	7.71 (d, 9.8)	143.6 (d)	7.71 (d, 9.8 )	143.6 (d)
5	7.20 (d,8.6)	122.3 (d)	7.36(d, 8.6)	123.0 (d)	7.36 (d, 8.6 )	123.0 (d)
6	6.98 (d,8.6)	109.3 (d)	6.97 (d, 8.6)	110.4 (d)	6.97 (d, 8.6)	110.4 (d)
7	/	153.5 (s)	/	154.3 (s)	/	154.3 (s)
8	/	135.6 (s)	/	136.3 (s)	/	136.3 (s)
9	/	113.4 (s)	/	114.1 (s)	/	114.1(s)
10	/	147.7 (s)	/	147.9 (s)	/	147.9 (s)
1'a	5.57 (s)	69.6 (t)	4.23 (m)	72.8 (t)	4.24 (m)	72.8 (t)
1'b			4.14 (m)	72.8 (t)	4.17 (m)	72.8 (t)
2'	/	193.8 (s)	4.66 (m)	72.7 (d)	4.68 (m)	72.6 (d)
3'	/	139.6 (s)	/	142.0 (s)	/	142.3 (s)
4'a	2.80 (dd,14.1, 4.8)	33.5 (t)	2.63 (dd, 14.6, 7.3)	36.4 (t)	2.69 (dd, 14.6, 5.1)	36.4 (t)
4'b	2.62 (dd, 14.1, 9.4)		2.54 (dd, 14.6, 6.4 )		2.45 (dd, 14.6, 8.1)	
5'	5.11 (m)	78.2 (d)	5.25 (m)	79.8 (d)	5.25 (m)	80.0 (d)
6'	7.01(d,1.6)	147.6 (d)	7.22 (d, 1.7 )	148.7 (d)	7.22 (d, 1.7 )	148.7 (d)
7'	/	128.8 (s)	/	130.0 (s)	/	130.0 (s)
8'	/	172.7 (s)	/	174.0 (s)	/	174.0 (s)
9'	1.77 (s)	9.3 (q)	1.98 (s)	10.5 (q)	1.98 (s)	10.5 (q)
10'a	6.37 (s)	128.3 (t)	5.43 (d, 6.8 )	116.2 (t)	5.45 (d, 6.8 )	115.9 (t)
10'b	6.09 (s)		5.28 (d, 6.8 )		5.28 (d, 6.8 )	
OMe	4.05 (s)	60.0 (q)	3.94 (s)	61.5 (q)	3.94 (s)	61.5 (q)

* Compound **1** recorded in pyridine-d_5_, **2**/**3** in CDCl_3_

The ^13^C-NMR spectrum (Table 1) showed, in addition to the resonances of the carbons belonging to the coumarin nucleus, 10 other signals arising from one methyl, one oxymethylene, one methylene, one oxygen-bearing methine, four olefinic and two carbonyl carbons that could only be located on the side-chain. Joint analysis of HSQC spectra and HMBC correlations (Figure 1) confirmed the presence of a C_10_ terpenoid side-chain attached to the coumarin skeleton. The connection of the side chain to C-7 on the coumarin nucleus was revealed by observation of a three-bond correlation between the oxymethylene proton signals (H-1') and carbon C-7 at δ_C_ 153.5s. The presence of only one methoxyl signal at δ_H_ 4.05 and a significant HMBC correlation with a quaternary carbon signal at δ_C_ 135.6s revealed that the methoxyl group must be unequivocally located on C-8. Meanwhile, the EI-MS spectra also showed characteristic 8-OMe coumarin fragment ion at *m/z* 193 corresponding to loss of side chain [10].

**Figure 2:**
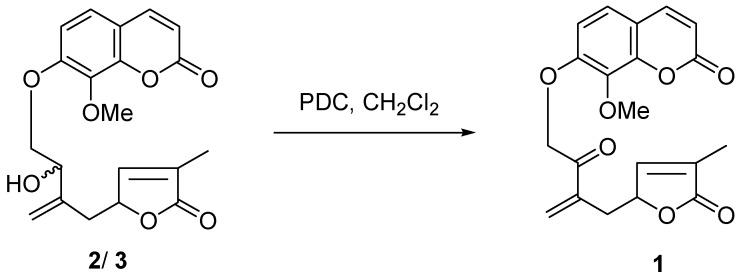
Oxidation of **2**/**3**.

Carefully analysis of the ^1^H and ^13^C NMR data of **1** (Table 1), showed that the signal patterns were in good agreement with those of previously isolated compounds **2**/**3** [13], except for some chemical shift differences. IR bands at 1,753, 1,748 and 1,730 cm^-1^ were indicative of the presence of three carbonyl groups in **1**, suggesting a ketonic group and a lactone in the side chain. The major difference between the ^13^C-NMR spectra of **1** and **2**/**3** was the appearance of a characteristic keto quaternary carbon signal at δ_C_ 193.8s (Table 1) in **1** and disappearance of oxygen-bearing carbons at δ_C_ 72.7d/72.6d compared to **2**/**3**. The proton signal at δ 5.57 (2H, H-1′) appeared as a singlet, revealing the ketone (*δ*_C_ 193.8s) was located at C-2′. In the HMBC spectrum (Figure 1), an informative correlation between the oxymethylene proton signal at δ_H_ 5.57 (2H, H-1′) and keto quaternary carbon signal at δ_C_ 193.8s confirmed it. Compared to **2**/**3**, because the C-2′ chiral center disappeared, there were no NMR signals pairs for C-2′, C-5′ and C-10′ in **1**. All these suggested that **1** was the C-2′ oxygenation product of **2**/**3**. To confirm the assigned structure, compound pair **2**/**3** was oxygenated to afford **1** with H_2_Cr_2_O_7_-2Pyr. (PDC) in CH_2_Cl_2_ (Figure 2). On the basis of these results, the structure of **1** as shown in Figure 1 is proposed. 

## Conclusions

As a continuation of our phytochemical investigation on *Clausena*, a new *O*-terpenoidal coumarin 1, named hekumarone, was isolated from the leaves and twigs of *Clausena anisum-olens* Merr., collected in Hekou County in Yunnan Province, P. R. China. It is documented that coumarins are one of the major constituents in plants belonging to the genus *Clausena* [4,5,6,9]. The result of the present study supports the conclusion that coumarins are characteristic and distinguishable chemical markers for the Rutaceae family, especially, the genus *Clausena*.

## Experimental

### General

Commercial silica-gel plates (Qing Dao Marine Chemical Group Co.) were used for TLC analyses. UV/VIS Spectra was measured on a Shimadzu UV-2401PC spectrophotometer; λ_max_ in nm. IR spectra were obtained on a Bio-Rad FTS-135 infrared spectrophotometer, ν_max_ in cm^-1^. ^1^H- and ^13^C-NMR as well as 2D-NMR spectra were recordered on a Bruker DRX-500 spectrometer with TMS as internal standard, coupling constant *J* are expressed in Hz. MS spectra were recorded on a VG Autospec-3000mass spectrometer.

### Plant material

The leaves and twigs of *Clausena anisum-olens* Merr. were collected in Hekou County of Yunnan province, P. R. China, in May 2003 and identified by Professor De-Ding Tao of the Kunming Institute of Botany. A voucher specimen (No. 02041705) is deposited in the State Key Laboratory of Phytochemistry and Plant Resources in West China, Kunming Institute of Botany, Chinese Academy of Sciences.

### Extraction and isolation

The powdered leaves and twigs of *Clausena anisum-olens* Merr. (22.5 kg) was repeatedly extracted with EtOH at room temperature. The extract was then concentrated under reduced pressure to give brown syrup, which was partitioned in H_2_O and extracted with solvents into petroleum ether-fraction, EtOAc-fraction and n-BuOH-fraction fractions. The EtOAc extracts (110.5g) were subjected to silica gel column chromatography eluting with PE-EtOAc (4:1, 2:1, 1:1, 2:3), EtOAc, EtOAc–MeOH (8:2, 7:3, 6:4, 1:1), MeOH, by which nine fractions (I-IX) were obtained. Fraction II was resubmitted to silica gel column chromatography, Pharmadex LH-20 (MeOH) and RP C-18 to yield compound 1 (4 mg). 

*Hekumarone* (**1**). Light yellow oil; ${\left[\alpha \right]}_{\text{D}}^{18.8}$– 6.67 (*c* 0.75, CH_3_OH); IR (KBr): IR ν^KBr^_max_ cm^-1^: 3,429, 2,923, 2,852, 1,753, 1,730, 1,606; UV (MeOH) λ_max_ nm: 319, 256, 208; ^1^H-NMR (δ ppm, pyridine-d_5_) and ^13^C-NMR data are shown in Table 1; EI-MS *m/z*: 370 ([M]^+^, 85), 273 (7), 204 (35), 193 (100), 161 (45); HRESI^-^MS *m/z* 369.1000 [M-1]^+^ (calcd. for C_20_H_18_O_7_ 369.0974).

### Oxidation of ***2**/**3***

PDC (60 mg) was resolved in dry CH_2_Cl_2_ (2 mL) under N_2_. The mixture was stirred 5 min at room temperature. Then a soln. of **2**/**3** (6 mg) in CH_2_Cl_2_ (1 mL) was added. The reaction mixture was stirred overnight. The mixture was filtered and H_2_O (2 ml) was added, and then extracted with CH_2_Cl_2_. The org. phase dried (MgSO_4_) and evaporated to afford **1** (3 mg) as a light yellow oil.
